# Drug therapy problems and predicting factors among ambulatory epileptic patients in Jimma Medical Center, Southwest Ethiopia

**DOI:** 10.1371/journal.pone.0267673

**Published:** 2022-04-28

**Authors:** Yadeta Babu Beyene, Fekede Bekele Daba, Kabaye Kumela Goro, Birbirsa Sefera Senbeta

**Affiliations:** 1 Department of Pharmacy, College of Health Science, Mettu University, Mettu, Oromia, Ethiopia; 2 School of Pharmacy, Institute of Health, Jimma University, Jimma, Oromia, Ethiopia; Cleveland Clinic, UNITED STATES

## Abstract

**Background:**

The care of epileptic patients is complicated by the cognitive adverse effect of the drug, disease, pharmacokinetics, and pharmacodynamics properties of antiepileptic drugs which in turn intensify the risk of drug therapy problems among epileptic patients.

**Objective:**

To assess drug therapy problems and predicting factors among ambulatory epileptic patients at Jimma University Medical Center, Southwest Ethiopia, from September 2020 to May 2021.

**Methodology:**

A hospital-based prospective observational study was conducted. A semi-structured questionnaire was used to collect data from patients as well as from charts. Drug therapy problems were identified using Cipolle’s, Morley, and Strand drug therapy problem identification and classification method. Data were entered into Epi data manager version 4.6 and exported to statistical software package for social science version 23.0 for analysis. Multiplestepwise backward logistic regression analysis was carried out to identify predictors of drug therapy problems. The 95% CI was used to show an association between the dependent and independent variables. P-value < 0.05 was considered as statistically significant.

**Results:**

Of the total 320 epileptic patients 224(70.0%) patients had at least one drug therapy problem. A total of 395 drug therapy problems were identified among two hundred twenty-four patients with an average of 1.2 drug therapy problems per patient. The frequently identified drug therapy problems were non-compliance 115(29.11), adverse drug reaction 110(27.84%), and dose too low 103(26%). Getting of a drug by purchasing [AOR = 4.6,95%CI:(2.05–10.7)], poorly involvement of the patients in therapeutic decision making [AOR = 3.02,95%CI:(1.5–6.06)], the number of medications ≥ two [AOR = 5.3,95%CI:(1.2–22.9)] and having had uncontrolled seizure [AOR = 10.9,95%CI:(4.9–24.2)] were independent predictors of drug therapy problems.

**Conclusions:**

Drug therapy problems were common among epileptic patients in the study area. Patients who were getting their drugs by purchasing, poorly involved in therapeutic decision making, having had an uncontrolled seizure, and taking two and above drugs were more likely to experience drug therapy problems. Therefore, due attention should be given to patients with the aforementioned problems to decrease the occurrence of drug therapy problems and improve overall outcomes among epileptic patients.

## 1. Background

Epilepsy is a chronic neurological disorder characterized by recurrent seizure attacks [1]. It accounts for 0.5% of the global burden of disease and has significant economic implications in terms of premature death, health-care-related needs, and lost work productivity [2]. In 2017 International League against Epilepsy (ILAE) classify epilepsy as focal seizures, generalized seizures, and unclassified seizures [3].

Epilepsy is a global public health problem affecting more than 70 million people worldwide, and more than 85% of the global burden of epilepsy occurs in developing countries [4]. In Africa, epilepsy affected about 10 million people and Ethiopia is one of the highly affected countries with an estimated prevalence of 14.2/1000 population and an annual incidence of 64 per 100,000 population [5].

Antiepileptic drugs (AEDs) are indicated for patients who have had one or more epileptic seizures. The choice of medication for the treatment of epilepsy is varied depending on the type, severity, and frequency of epileptic seizures [6, 7]. The majority of epileptic seizures are controlled with the optimal use of the currently available AEDs. However, about one-third remained uncontrolled despite optimal therapy [8, 9] Drug therapy problem (DTP) is common in epileptic patients because of the complexity of AEDs and the disease itself. The complexity of medical problems and comedications for patients requiring AEDs can result in an increased likelihood of drug interactions and drug toxicity which in turn intensify the risk of having drug therapy problems and negatively affects the seizure control status of the patients [10].

DTP is an event related to medication therapy that actually or potentially interferes with the desired health outcome [11]. DTP can occur at various stages of the medication use processes, including while prescribing, transcribing, dispensing, and when the patients use drug therapy [12]. DTPs are categorized into seven major classes composed of, unnecessary drug therapy, need additional drug therapy, ineffective drug therapy, dosage too low, dosage too high, adverse drug reaction (ADR), and non-compliance [12].

Generally, DTP is one of the major public health problems and has been significantly increased over the past few years. Despite more than 50% of DTP is being avoidable 5–10% of hospital admission were due to DTP [13]. The World Health Organization (WHO) estimates that globally “more than half of all medicines are prescribed, dispensed, or sold inappropriately and that half of all patients fail to take their medicines correctly [14, 15]. In the US alone it is estimated that 100,000 deaths occur annually due to drug therapy problems and costing approximately $201.4 billion in direct medical costs per year [16].

DTPs in epileptic patients is among major factors that affect the outcome of therapy and the safety of medical care. In epileptic patients, the frequency of DTP has been reported to be as high as 70% to 90.38%, [17, 18]. Disregard to such high-frequency previous studies done in Ethiopia among epileptic patients was primarily focused on the assessment of adherence, but there is only limited study done on the prevalence of drug therapy problem, type of drug therapy problem, and predicting factors of drug therapy problems among epileptic patients. Consequently, this study aimed at assessing drug therapy problems and its predicting factors among epileptic patients at the ambulatory clinic of Jimma University medical center and providing clinical pharmacist intervention for identified drug therapy problems.

## 2. Methods and materials

### 2.1 Study design and study setting

A hospital-based prospective observational study was conducted from September 2020 to May 2021 at the ambulatory care clinic in JUMC, Oromia, Ethiopia. JUMC is located in Jimma town, 352 km Southwest of Addis Ababa, Ethiopia. It is among the largest teaching institution in Ethiopia and is the only teaching and referral hospital in the southwestern part of the country and provides services for the catchment population of about 15 million people.

### 2.2 Study population and data collection procedure

Adult epileptic patients with complete medical records and regular follow-up for minimum of six months were included in the study. Patients were included into the study during their appointment for medication refilling. Patients who were, seriously ill to complete the interview, unwilling to give consent, and had incomplete medical records were excluded from the study. A sample of 320 was calculated using a single population proportion formula by using the following assumptions: Z = 1.96, P = 70.4% proportion of DTP at TASH [17], 95% confidence level, 5% margin of error. Patients were interviewed during their follow-up period for the medication refilling and the medical chart was retrieved using the checklist for retrieval. Interview questions included socio-demographics, behavioral-related factors, patient involvement in therapeutic decision making, and noncompliance assessment. The respective patient’s medical and medication records were reviewed. Three clinical pharmacists (1^st^-degree holders) were involved in the data collection process. One day training and orientation were given to the data collectors before the beginning of the study.

### 2.3 Drug therapy problems identification and classification

In the current study, DTPs were classified according to Cipolle, Morley, and Strand’s DTPs classification method [12]. The DTPs were identified by an independent team of clinical pharmacists by using the data collected from the patients through interviewing and medical records through reviewing. The prescribed drugs were evaluated against different international and national guidelines such as National Institute for Health and Care Excellence (NICE), American Academy of Neurology 2018 (AAN), ILAE guideline, Dipro-pharmacotherapy 11th edition, Epilepsy Prescriber’s Guide 3rd edition, 2020 Ethiopian standard treatment guideline, UpToDate 2018 and Lexicomp version 1.1 drug interaction checker. The identified DTP were classified as, need additional drug therapy, too low dosage, ineffective therapy, too high dosage, ADR, and non-compliance.

### 2.4 Intervention development and implementation

Pharmacist’s interventions were provided for individual patients based on the types of identified DTPs. If the identified DTP was non-compliance, counseling was provided for the patients on spot. For other types of DTP requiring the involvement of physicians, the clinical pharmacists were communicated with the responsible physician and provided recommendations with the ultimate goal of optimizing medication therapy by using the aforementioned reference materials on the patient’s appointment day for a refill.

### 2.5 Data quality assurance

Pretest was done on sixteen patients, (5%) of the study participants to ensure the appropriateness of the data collection instruments, and the modifications were undertaken based on the information from pre-test. The data collection processes were supervised and the collected data were checked by the principal investigator (PI) on the daily basis.

### 2.6 Statistical analysis

The collected data were compiled, cleared, coded, and checked for completeness and accuracy before entering into software. Epi data manager version 4.6 and SPSS version 23.0 were used for data entry and analyses respectively. All statistical tests were performed using SPSS version 23.0. Descriptive statistics were computed as frequency, mean and standard deviation (SD). Multicollinearity was checked among independent variables and no collinearity was found. To determine the association between dependent and independent variables, Univariable logistic regression analysis was done. The independent variables with a p-value <0.25 were included in a final model (multivariate) to identify predictors of DTPs. A p-value of <0.05 was considered statistically significant in all analyses. The Odds ratio with a 95% confidence interval (CI) was used to measure the strength of association between independent and dependent variables. The outputs of processed data were presented by tables and figures.

### 2.7 Ethical consideration

The approval letter for this study was taken from the institutional review board (IRB) of Jimma University, College of Health Sciences. The objective of the study was explained and written informed consent was obtained from study participants. For the matter of patient confidentiality, the name and address of the patients were not included in the data collection tools.

### 2.8 Operational definitions

**Controlled seizure:** is free of seizure episode for the minimum of the last two years.

**Uncontrolled seizure:** Patients experienced at least one seizure episode in the last two years.

**Unclassified seizure:** Documented diagnosis as epilepsy disorder without any classification.

**DTP:** DTP is an event related to medication therapy that actually or potentially interferes with the desired health outcome [11].

**ADRs**: Any noxious, unintended, and undesired effect experienced by the patient which was associated with the drug.

**Co-morbidity:** Medical condition/s other than epilepsy.

**Pharmacist intervention**: A recommendation suggested by the clinical pharmacist for changing the patients’ therapeutic management.

**Accepted:** When the suggested problems were recognized and accepted.

**Drug interaction**: Presence of significant drug-drug interaction.

**Patient involvement in drug therapy decision**: This is the degree of patient involvement in the making of decision about their drug therapy. It was measured using a 2-item 5-point Likert scale using a self-reported questionnaire about patient involvement in therapeutic decision making. The respondents indicated their degree of involvement ranging from 1 = never to 5 = all times. Accordingly, patients were said to be actively involved if the average score was above the mid-point (*>*5) otherwise poorly involved [19]. The identified DTPs were categorized based on Cipolle’s method [12] as follows:

**Need for additional drug therapy**: was considered when the medical condition requires additional drug therapy to achieve synergistic or additive effects.

**Ineffective drug therapy**: was considered when the drug is used for a medical condition that is refractory to the drug product.

**Dosage too high**: was considered when the dose is too high, the duration of drug therapy is too long and the drug interaction occurs that could result in a toxic reaction.

**Too low dose**: was identified when the prescribed dose was too low to produce the intended response, and when the amount of active drugs available were reduced due to drug interaction.

**Adverse drug reaction**: was considered when the drug causes an undesirable reaction that was not dose-related, a safer drug product is required due to risk factor, the drug product is contraindicated due to risk factor.

**Noncompliance**: was considered if a patient fails to take medications appropriately due to one of the following reasons, the difficulty of understanding the instructions, preferring not to take medication, expensiveness of drug product, forgetfulness, and unavailability of medication.

## 3. Results

### 3.1 Socio-demographic and behavioral related characteristics

A total of 320 epileptic patients were included in the study. The study subject’s mean age was 29.9 ±11.2 SD and about half of them, 149(46.6%) were young adults within the age category of (18–25 years). More than half of the study subjects were male 176(55%) and 170(53%) of study participants were from rural areas. Regarding monthly income 287(89.7%) of study, subjects have no regular income. Among the study participants, 230 (71.9%) were educated, and greater than half, 188 (58.8%) of the study participants were getting their medication freely. From study participants, only 64(20%) were social drug users (Table 1).

**Table 1:** Socio-demographic and behavioral related characteristics of epileptic patients attending the ambulatory clinic of JMC, 2021.

Characteristics	Frequency (n)	Percentage (%)
Mean (±SD) age		29.9 ±11.2
Age in years		
18–25	149	46.6
26–44	132	41.3
≥45	39	12.2
Gender		
Male	176	55
Female	144	45
Residency		
Urban	150	46.1
Rural	170	53.1
Monthly income		
No regular income	287	89.7
Regular income	33	10.31
Educational status		
Un educated	90	28.1
Educated	230	71.9
Occupation		
Farmer	87	27.2
Student	60	18.8
Daily laborer	29	9.1
Employed	22	6.9
Merchant	14	4.4
Housewife	36	11.3
Jobless	72	22.5
Religion		
Orthodox	77	24.1
Muslim	218	68.1
Protestant	20	6.3
Catholic	5	1.6
Living condition		
Living alone	22	6.9
With family	298	93.1
How to get medication		
Free	188	58.75
Payment	132	41.25
Social drug-using status		
Yes	64	20
No	256	80
Patient involvement		
Actively involved	117	36.56
Poorly involved	203	63.43

### 3.2 Clinical characteristics of the study participants

From the study participants, 221 (69.1%) were diagnosed with generalized tonic-clonic seizure(GTC). Hundred sixty-two (50.6%) of the patients had a follow-up duration lasting >10 years and half of the study participants 168(52.5%) had controlled seizure. Regarding the comorbid medical condition, 69(21.6%) of participants had comorbidity and frequently reported comorbid medical conditions were dyspepsia 19(5.9%) and hypertension 15(4.6%) (Table 2).

**Table 2:** Clinical characteristics of epileptic patients attending ambulatory clinic of JMC, 2021.

Clinical characteristics	Frequency (n)	Percentage (%)
Types of seizure		
GTC	221	69.1
Focal	25	7.8
Unclassified*	74	23.1
Follow-up duration		
1–5 years	80	25
6–10 years	78	24.4
>10 years	162	50.6
Seizure control status		
Controlled	168	52.5
Uncontrolled	152	47.5
Comorbidity		
Yes	69	21.6
No	251	78.4
Comorbid condition		
Dyspepsia	19	5.9
Hypertension	15	4.6
Schizophrenia	7	2.2
PNP	5	1.5
RVI	4	1.3
DM	4	1.3
Hemorrhoid	4	1.3
Back pain	3	0.9
Stroke	3	0.9
Others^a^	5	1.5

PNP: Peripheral neuropathy, RVI: Retroviral Infection, DM: Diabetic Mellitus, GTC: Generalized tonic-clonic, Unclassified*: documented diagnosis as epilepsy disorder without any classification, Others^a^: Ischemic heart disease, Sexual dysfunction, Tinea pedis, Migraine headache.

### 3.3. Antiepileptic drug uses pattern among the study participants

A total of 494 AEDs were prescribed over the study period with an average of 1.54 ± 0.5 SD AEDs per patient. Half of the study participants, 162(50.6%) were on a single therapy regimen. Phenobarbital was the most frequently prescribed drug as monotherapy and combination therapy with phenytoin,117(36.5%) and 107 (33.4%), respectively. Regarding the total number of drugs only 50(15.6%) of participants were taking three and above drugs (Table 3).

**Table 3:** Medication use pattern of epileptic patients attending ambulatory clinic of JUMC, 2021.

Variables	Frequency (n)	Percentage (%)
Number of AED		
One	162	50.6
Two	142	44.3
Three	16	5
AED use pattern		
PB	117	36.5
PHT	39	12.2
VPA	4	1.3
CBZ	2	0.6
PB+PHT	107	33.4
PB+CBZ	25	7.8
PB+VPA	3	0.9
PHT+CBZ	4	1.3
PHT+VPA	3	0.9
PB+PHT+CBZ	11	3.4
PB+PHT+VPA	3	0.9
PHT+CBZ+VPA	2	0.6
Total drugs per patient		
One drug	137	42.8
Two drugs	133	41.56
Three drugs &above	50	15.6

PHB: phenobarbital, PHT: phenytoin, CBZ: carbamazepine, VPA: Valproic acid.

### 3.4 Types, prevalence, and cause of drug therapy problems

**Fig 1:**
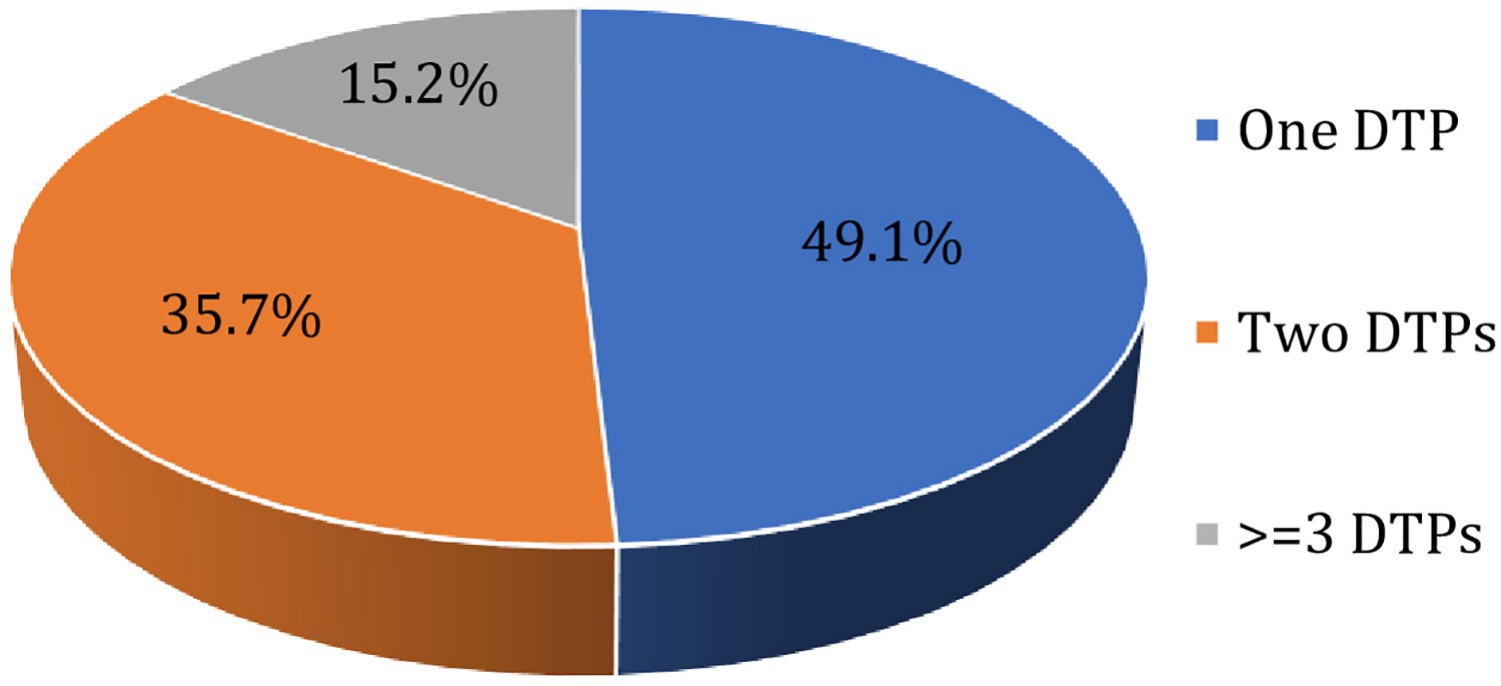
Number of drug therapy problems among epileptic patients attending the ambulatory clinic of JMC, 2021.

From a total of 320 patients, 224 patients experienced DTPs, with an overall prevalence of 70%. The most frequent DTPs were noncompliance 115(29.1%), ADR 110(27.8%), and too low dosage 103(26%) (Table 4). From 224 patients who had experienced DTPs, about half of the patients 110(49.1%) had one DTP, 80 (35.7%) patients had two DTPs and 34(15.2%) had three and above DTPs. The average number of DTP per patient was 1.2 (Fig 1).

**Table 4:** Types and cause of DTP among epileptic patients at the ambulatory clinic of JMC, 2021.

DTP category and causes	Proportion of DTP, n = 395
**Noncompliance**	**115(29.1)**
Does not understand the instruction	2(1.7)
Prefer not to take the medication	11(9.6)
The drug product is too expensive for the patient	33(28.7)
Forgets to take medication	39(33.9)
The drug product is not available for the patient	30(26.1)
**ADR**	**110(27.8)**
The drug product causes an undesirable reaction that is not dose-related	97(88.2)
A safer drug product is required due to risk factors	7(6.4)
The drug product is contraindicated due to risk factors	6(5.4)
**Too low dosage**	**103(26)**
Th**e** dose is too low to produce the desired response	64(62.1)
A drug interaction reduces the amount of active drugs available	39(37.8)
**Too high dosage**	**55(13.9)**
The dose is too high	44(80)
The duration of drug therapy is long for a given condition	6(10.9)
Drug interaction	**5(9)**
**Additional drug therapy**	**9(2.2)**
To attain synergistic effect or additive effect	9(100)
**Ineffective drug therapy**	**3(0.76)**
The medical condition is refractory to the drug	3(100)

### 3.5 The types and status of interventions carried out

A total of 358 (90.6%) interventions were undertaken by clinical pharmacists. The remaining (4.3%) were solved by the other health care team and (5.1%) were not intervened due to loss of patient from follow-up. Counseling of the patients (58.94%) and recommendation for dosage adjustment (30.97%) were the most common intervention provided by clinical pharmacists during the study period. From the total intervention given, 254(70.94%) were accepted fully with an acceptance rate of 71% (Table 5).

**Table 5:** Types and status of intervention provided for epileptic patients attending ambulatory clinic of JMC, 2021.

Types and status of intervention	Frequency (n)	Percentage (%)
Counseling on adherence and ADR	211	58.94
Increasing dosage	61	17
Decreasing dosage	50	13.97
Addition of medication	7	1.96
Switching of medication	27	7.54
Discontinuation of medication	2	0.56
Intervention is fully accepted	254	71
Intervention is accepted with modification	43	12.01
Intervention is not accepted	61	17.04

### 3.6 Predicting factors of drug therapy problems

Depending on the outputs of multivariable logistic regression model getting of medications by purchasing (AOR = 4.6,95%CI = 2.05–10.7), Poorly involvement of the patient in therapeutic decision making (AOR = 3.02,95%CI = 1.5–6.06), Having had an uncontrolled seizure (AOR = 10.9,95%CI = 4.9–24.2), and taking of two and above drugs (AOR = 5.3,95%CI = 1.2–22.9) were independent predicting factors of DTPs (Table 6).

**Table 6:** Bivariate and multivariate analysis of factors associated with DTPs among epileptic patients attending ambulatory clinic of JMC, 2021.

Variables	DTP		Bivariate analysis		Multivariate analysis	
Age in year	Yes	No	COR	P-value	AOR	P-value
18–25	97(43.3)	52(54.17)	1		1	
26–44	98(43.8)	34(35.4)	1.54(0.92–2.5)	0.098	1.46(0.68–3.1)	0.323
≥45	28(12.5)	11(11.45)	1.36(0.63–2.96)	0.432	1.15(0.39–3.3)	0.793
Residency						
Urban	111(49.6)	39(40.6)	1		1	
Rural	112(50.4)	58(60.4)	0.67(0.41–1.1)	0.116	0.98(0.48–1.9)	0.98
Social drug use status						
Yes	42	22	0.77(0.43–1.39)	0.394	------------------------	---------------
No	182	74	1			
Medication fee						
Free	103(45.9)	85(88.5)	1		1	
Payment	120(53.5)	12(12.5)	8.2(4.2–15)	<0.001	4.6(2.05–10.7)	<0.001
Patient involvement						
Actively involved	56(25)	61(63.5)	1		1	
Poorly involved	167(74.5)	36(37.5)	3.3(2–5.5)	<0.001	3.02(1.5–6.06)	0.002
Type of seizure						
GTC	158(70.5)	63(65.6)	1			
Focal	19(8.4)	6(6.25)	1.2(0.48–3.3)	0.635	0.47(0.12–1.9)	0.293
Unclassified	46(20.5)	28(29.1)	0.65(0.37–1.13)	0.134	0.41(0.11–1.7)	0.24
Follow up duration						
1–5 years	49(21.8)	32(33.33)	1		1	
6–10 years	56(25)	22(22.9)	1.7(0.85–3.2)	0.134	1.74(0.68–4.4)	0.244
>10 years	118(52.6)	43(44.8)	1.8(1.02–3.15)	0.043	1.6(0.71–3.78)	0.243
Seizure control status						
Controlled	82(36.6)	86(89.58)	1		1	
Uncontrolled	141(62.9)	11(11.45)	13.4(6.7–26.6)	<0.001	10.9(4.9–24.2)	<0.001
Quantity of AED						
One	96(42.85)	64(66.7)	1			
Two and above	127(56.7)	33(34.37)	2.5(1.5–4.2)	<0.001	0.29(0.06–1.4)	0.129
Comorbidity						
Yes	61(27.23)	8(8.3)	4.2(1.9–9.1**)**	<0.001	1.73(0.61–4.8)	0.297
No	162(72.3)	89(92.7)	1		1	
Total drugs						
One	75(33.48**)**	62(64.58**)**	1		1	
Two	100(44.6)	33(34.37)	2.5(1.5–4.2)	0.001	5.3(1.2–22.9)	0.024
Three and above	48(21.42)	2(2.08)	19.8(4.6–24)	<0.001	31(3.8–50)	0.001

## 4. Discussion

In clinical practice, a wide range of DTPs may arise [20]. The prevalence of DTPs among epileptic patients varied from study to study. In this study setting the prevalence of DTPs was found to be 70%, which was in line with the study conducted in TASH (70%) [17] and in Bangalore (76.9%) [21], but higher than the study reported from China (48.1%) [22] and India (13%) [23]. This variation might be due to the variation of clinical characteristics of study participants, availability of trained prescribers, and methods of DTP identification and classification.

In the current study, the frequently identified DTP was noncompliance 115(36%) with the main reasons of forgetfulness followed by the expensiveness of the drug products. This finding was comparable with the other study done in Ethiopia (34.1%) [24], Saud Arabia (38%) [25], India (37%) [26], and China (33.3%) [27]. However, it was higher than the study conducted in Paris [28] and USA [29] where 21% and 26% of study participants were noncompliant to AEDs respectively. The probable reasons for this discrepancy could be due to differences in economic status, availability of drugs, belief, education, and physicians’ approach to their patients that could bring differences in patients’ attitudes towards medications.

Adverse effects of AEDs are major causes of iatrogenic disease and make a significant contributionto reduced quality of life in individuals with epilepsy. Overall, the incidence of patients experienced ADR in this study was found to be 110(34.37%) which was in line with the finding reported from Malaysia (31%) [30], India (30%) [26], and Ethiopia (37%) [24]. This showed that adverse effects associated with AED were common among epileptic patients. Therefore, its assessment should be routine practice at each visit.

In this study, inappropriate dosing (dose too low and dose too high) accounted for 40% of allDTPs. This finding was two times higher than the study reported from Bishoftu general hospital(17.6%) [31] and Thailand (19.47%) [18]. This variation could be due to differences in the DTP classification method used, less frequent use of combination therapy, and short duration of study period by the previous study.

In this finding, 41.25% of study participants were getting their medication through purchasing and DTPs were more likely to happen among this group of patients than those patients who were getting their medications freely. This finding was in agreement with a study done in Kenya [32] and Dilla University Referral Hospital [33], where a similar group of patients had three times and two times chance of developing DTP respectively. The possible explanations for why these groups of patients were more prone to have DTP could be due to the shortage of the resources they were not compliant to their medications and follow-up schedule which increases the risk of having DTPs among these groups of patients.

The other independent predicting factor for the occurrence of DTP among study participants was having had an uncontrolled seizure. In this finding, 47.5% of the study participants had an uncontrolled seizure and DTPs were more common in this group of patients than patients with controlled seizures. This finding was in line with former studies from Brazil [34], Indonesia [35], and Bangladesh [36], where DTP was highly common among a group of patients with an uncontrolled seizure. This could be due to patients with uncontrolled seizures may have a wrong attitude about their medication’s effectiveness and may refuse to take it. Therefore, further recognition and support should be given to patients with uncontrolled seizures since they are more likely to be more anxious and may have wrong beliefs about their medications.

A number of drugs were one of the other independent predicting factors for DTPs. Accordingly, those patients who were taking two and above medications were more prone to have DTPs than patients who were taking less than two medications. This finding was augmented by the study reported from Mekelle University [37], Saud Arabia [25], and Nigeria [38], where multiple therapies significantly influence the occurrence of DTPs. The possible reason might be since, multiple therapies are attributed to the complexity of a treatment regimen involving a larger number of pills that need to be taken at different time intervals, which increases the likelihood of missed doses, drug interaction, and ADR. Hence health care professionals should give due attention to those patients to decrease the likelihood of DTP occurrence and improve treatment outcomes among epileptic patients.

Active participation of patients in their care process is important to provide quality of pharmaceutical care service and in minimizing problems associated with the treatment [12]. In this study patients who were poorly involved in their drug therapy decision making were more likely to have DTPs than those who were actively involved in decision-making. This finding was supported by the study conducted in other chronic medical conditions at JUMC, in which poorly involved patients were four times more likely to have DTPs than actively involved patients [19]. The possible explanations for this could be poorly involved patients in therapeutic decision making may have a wrong belief about their disease and medication which make them nonadherent to their AED. Therefore, to minimize the occurrence of DTP, the involvement of patients in decision making needs to be part of the management process in epileptic patients.

In the current study, a total of 358 clinical pharmacist interventions were provided at the level of patients and physicians with an acceptance rate of 71%. This finding was supplemented by another study from Goba Referral Hospital, in which (72.6%) interventions were accepted [39]. In this finding counseling of the patients (59%) and dosage change (31%) were the top two clinical pharmacist interventions provided for epileptic patients during the study period. This showed that clinical pharmacists can prevent and resolve DTPs through effective communications and collaboration with other health care professionals and patients.

## 5. Conclusion

Drug-related problems were prevalent among ambulatory epileptic patients on follow-up during the study period. The most frequently identified DTPs were non-compliance, ADR, and inappropriate dosage. The finding also revealed that source of medication fee, number of drugs, patient involvement in therapeutic decision making, and seizure control status were independent predicting factors of DTPs. Regarding the intervention given, counseling of patients and dosage adjustments were the top two clinical pharmacist’s interventions provided with an overall acceptance rate of 71%. This highlights the role of clinical pharmacists in optimizing therapy among epileptic patients.

## Supporting information

S1 File (DOCX)

S1 Dataset (SAV)
