# Supplementary material for: Drug therapy problems and predicting factors among ambulatory epileptic patients in Jimma Medical Center, Southwest Ethiopia
Source: PLoS One. 2022 Apr 28;17(4):e0267673. doi: 10.1371/journal.pone.0267673 (PMC9049505; doi:10.1371/journal.pone.0267673)
Supplement: S1 File — (DOCX) [file pone.0267673.s001.docx]

**Jimma University**

**College of Health Sciences**

**School of Pharmacy**

**Data collection tool to identify drug therapy problems and predicting factors among ambulatory epileptic patients in JMC.**

**Questionnaire English Version**

Part I: Participant’s Socio demographic characteristics

| 1.Card no.______ | 2.Age(years): ______ | 3.Gender  a) Male  b) Female | | 4.Residency  a) Urban  b) Rural | 5.Monthly income  a) No regular income  b) -------ETB/Month |
| --- | --- | --- | --- | --- | --- |
| 6.Educational status  a) Un educated  b) Primary education (grade 1-8)  c) Secondary education (9-12 grade)  d) Tertiary education (diploma & above) | | 7.Occupation  a) Farmer  b) Student  c) Daily laborer  d) Employed  e) Merchant | | | 8.Religion  a) Orthodox  b) Muslim  c) Protestant  d) Catholic |
| 9.Living condition  a) Living alone  b) With family | | 10.Source of medication fee  a) Free  b) Payment | | |  |
| **Part II: Behavioral related factors** | | | | | |
| 1. Alcohol consumption  a) Yes  b) No | | | 2. Cigarette smoking  a) Yes  b) No | | 3. Khat chewing  a) Yes  b) No |
| **Part III: Assessment of patient involvement in drug therapy decision making** | | | | | |
| 1. Did you get involved in choosing your treatment and care?  a) All-time b) Most of the time c) Some times d) Rarely e) Never  2. Were you given information about options for choosing a treatment appropriate for your problems  a) All-time b) Most of the time c) Sometimes d) Rarely e) Never | | | | | |

**Part IV: Clinical characteristics (supplementary to the information obtained from medical chart)**

1**.** When did the first seizure episode occur? _______________

2**.** Time of epilepsy diagnoses? __________________________

3. When did you encountered the recent seizure episode? _______________

4. When did you start your AEDs? ______________________

5. How many AEDs you are taking currently? __________

6. Diagnosis other than epilepsy (comorbidity): _________________

7. Total number of medications you are taking currently? ___________

**Part V: Data abstraction format from patient medical records**

1.Diagnosis of seizure type: ________________________
2. Diagnosis other than epilepsy (comorbidity): _____________

| 3. **Medication history, history of seizure episode and ADR report on consecutive visits** | | | | | | | | |
| --- | --- | --- | --- | --- | --- | --- | --- | --- |
| First visit | | | Second visit | | | Third visit | | |
| Medication | Seizure episode | ADR | Medication | Seizure episode | ADR | Medication | Seizure episode | ADR |
|  |  |  |  |  |  |  |  |  |
|  |  |  |  |  |  |  |  |  |
|  |  |  |  |  |  |  |  |  |
|  |  |  |  |  |  |  |  |  |
|  |  |  |  |  |  |  |  |  |

**4. Current and previous medication**

| For the current medical diagnosis (including Comorbid and complications) | | | | |
| --- | --- | --- | --- | --- |
| Medical condition/ Indication | Product name (Generic name) | Dosage regimen (dose, route, frequency, duration) | Previous medication, if changed | Response Effectiveness/ safety profile |
|  |  |  |  |  |
|  |  |  |  |  |
|  |  |  |  |  |

5. Is there any drug interaction? a) Yes b) No

6. Was there any experienced adverse effects of the drugs? a) Yes: b) No

**Part VI: Assessment of DTPs of the patient**

1. Is there a **need for additional drug therapy**? a) Yes b) No

2. If yes for no. 1, what is the reason for additional drug therapy need?

a) A medical condition requires initiation of drug therapy

b) Preventive drug therapy required to reduce the risk of developing a new condition.

c) To attain synergistic effect or additive effect

3. If” yes” for number,1 please list those medical problems needing additional medication?

| Date | Indication | Recommended drug regimen | Cause (write letters) |
| --- | --- | --- | --- |
|  |  |  |  |
|  |  |  |  |

4. Is there any **unnecessary drug** therapy for the patient? a) Yes b) No

5. If yes for no. 4, what are the reasons for unnecessary drug therapy?

a) No valid medical indication for the drug therapy at this time

b) Multiple drug products are used for a condition that needs single-drug therapy.

c) The medical condition is more appropriately treated with non-drug therapy.

d) Drug therapy is used to treat an avoidable ADR associated with a drug

e) Drug abuse, alcohol use, or smoking is causing the problem

6. If” yes” for number,4 please list the unnecessary prescribed medication and causes?

| Date | Indication | Drug regimen with problem | Cause (write letters) |
| --- | --- | --- | --- |
|  |  |  |  |
|  |  |  |  |

7. Is there **any ineffective drug therapy** used? a) Yes b) No

8. If yes for no.7, what was the cause?

a) The drug is not the most effective for the medical problem b) The medical condition is refractory to the drug

c) The dosage form of the drug product is inappropriate

d) The drug product is not an effective product for the indication being treated

9. If yes for number 7 list the ineffective medication used?

| Date | Indication | Drug regimen with problems | Cause (write a letter) |
| --- | --- | --- | --- |
|  |  |  |  |
|  |  |  |  |

10. Is there any medication with **too low a dosage**? a) Yes b) No

11. If yes for number.10. What was the cause for the dosage being too low?

a) The dose is too low to produce the desired response.

b) The dosage interval is too infrequent to produce the desired response.

c) A drug interaction reduces the amount of active drugs available.

d) The duration of drug therapy is too short to produce the desired response.

12. If yes for number 10 list those doses too low with their causes?

| Date | Indication | Drug regimen with problems | Cause (write letters) |
| --- | --- | --- | --- |
|  |  |  |  |
|  |  |  |  |

13. Is there any medication with **too high dosage**? a) Yes b) No

14. If yes for question number.13 what is the cause for the dosage to be high?

a) Dose is too high b) The dosing frequency is too short.

c) The duration of drug therapy is long for a given condition.

d) A drug interaction occurs resulting in a toxic reaction to the drug product.

e) The dose of the drug was administered too rapidly f) Adjustment for renal impairment was not done

15. If yes for question number 13. Please list those with doses too high with their causes

| Date | Indication | Drug regimen with problems | Cause (letters) |
| --- | --- | --- | --- |
|  |  |  |  |
|  |  |  |  |

16**.** Is there **any adverse drug reaction**? a) Yes b) No

17. If “yes” for number 16 what was the cause for the ADR?

a) The drug product causes an undesirable reaction that is not dose-related.

A safer drug product is required due to risk factors.

c) A drug interaction causes an undesirable reaction that is not dose-related.

d) The drug product causes an allergic reaction. e) The drug product is contraindicated due to risk factors

| Date | Indication/s | Drug regimen with problems | Cause (letters) |
| --- | --- | --- | --- |
|  |  |  |  |
|  |  |  |  |

18. Is there any **compliance problem**? A) yes B) no

If yes what could be the possible causes for non-compliance?

a) Does not understand the instruction b) Prefer not to take the medication.

c)The drug product is too expensive for the patient. d) Forgets to take medications

e) Cannot swallow or self-administer the drug product appropriately.

f) The drug product is not available for the patient

**Part VII: Table of DTP Summary**

| **S.no** | **Type of DTP** | **Cause of DTP** | **Intervention given** | **Intervention by** | **Status of intervention** | **Remarks** |
| --- | --- | --- | --- | --- | --- | --- |
|  |  |  |  |  |  |  |
|  |  |  |  |  |  |  |
|  |  |  |  |  |  |  |
|  |  |  |  |  |  |  |
|  |  |  |  |  |  |  |
|  |  |  |  |  |  |  |
|  |  |  |  |  |  |  |
|  |  |  |  |  |  |  |
|  |  |  |  |  |  |  |

**Part VIII. Acceptance and Implementation of intervention (tick one box only)**

**1. Intervention accepted 2. Intervention not accepted**

a**.** Intervention is fully accepted a. Due to lack of physician cooperation

b. Intervention accepted with modifications b. Due to lack of patient cooperation
